# Shared reading is associated with fewer emotional/behavioral problems and better prosocial behavior in preschool children: a cross-sectional study in western China

**DOI:** 10.3389/fpsyt.2026.1858077

**Published:** 2026-06-24

**Authors:** Hongli Sun, Huifang Zhang, Huiping Zhang, Yudan Zhang

**Affiliations:** 1 Department of Sociology, School of Humanities and Social Sciences, Xi’an Jiaotong University, Xi’an, Shaanxi, China; 2 Shaanxi Institute for Pediatric Diseases, Xi’an Key Laboratory of Children’s Health and Diseases, Xi’an Children’s Hospital, Affiliated Children’s Hospital of Xi’an Jiaotong University, National Regional Children’s Medical Center (Northwest), Xi’an, Shaanxi, China; 3 Department of Emergency, Xi’an Children’s Hospital, Affiliated Children’s Hospital of Xi’an Jiaotong University, National Regional Children’s Medical Center (Northwest), Xi’an, Shaanxi, China; 4 Neonatal Intensive Care Unit, Xi’an Children’s Hospital, Affiliated Children’s Hospital of Xi’an Jiaotong University, National Regional Children’s Medical Center (Northwest), Xi’an, Shaanxi, China

**Keywords:** emotional/behavioral problems, home literacy environment, preschool children, prosocial behavior, shared reading, StimQ-P, strengths and difficulties questionnaire (SDQ)

## Abstract

**Background:**

The home literacy environment, particularly shared reading, plays a critical role in preschool children’s cognitive and socioemotional development. However, its associations with emotional and behavioral problems remain underexplored in large-scale studies. This study examined the relationship between shared reading and emotional/behavioral problems as well as prosocial behavior in preschool children.

**Methods:**

A cross-sectional study was conducted using stratified cluster sampling across 189 kindergartens in a major city in western China. A total of 21,366 parent-child pairs were included. Shared reading was assessed with the reading subscale of the StimQ-P (score range 0–22), which evaluates quantity, diversity of concepts and content, and interactivity quality. Emotional and behavioral problems were measured using the parent-reported Strengths and Difficulties Questionnaire (SDQ). Multivariate logistic regression and generalized additive models were employed to examine associations, adjusting for child age, gender, parental socioeconomic factors, lifestyle variables, and parental mental health (CES-D).

**Results:**

Higher shared reading scores were significantly associated with lower odds of emotional/behavioral problems (adjusted OR = 0.96 per point increase, 95% CI: 0.95–0.97, P < 0.0001) and higher odds of adequate prosocial behavior (adjusted OR = 1.09, 95% CI: 1.08–1.10, P < 0.0001) in fully adjusted models. All four dimensions of shared reading showed independent associations. Nonlinear analyses revealed threshold effects, with associations becoming stronger above approximately 18 points for total difficulties and 15 points for prosocial behavior. These associations were largely consistent across subgroups after correction for multiple testing.

**Conclusion:**

In this large cross-sectional study conducted in western China, higher levels of shared reading were associated with lower odds of emotional/behavioral problems and higher odds of prosocial behaviors among preschool children. The results suggest possible threshold patterns in these associations. However, given the cross-sectional nature of the study, causality cannot be established.

## Introduction

The shared reading encompasses a wide range of factors, such as the availability of reading materials, opportunities for shared and independent literacy activities, and the support children receive from family members in their learning experiences (1, 2). In this article, “shared reading” is evaluated using the preschool version of StimQ-P, which measured four reading-related aspects: quantity, diversity of concepts, diversity of contents, and interactivity quality (3, 4). A large body of research has shown that shared reading contributes to preschool children’s positive development, including more expressive vocabulary (5), better conceptual vocabulary skills (6), development of language (7), executive functions abilities and cognitive abilities (3, 8). The benefits of a rich shared reading during the preschool years may extend into primary school and adulthood (9–11). Early vocabulary development was positively correlated with early social-emotional and behavioral development in children (12).

Recent research has additionally shown that a richer shared reading is directly associated with better emotional regulation in children (13). Shared reading has been empirically validated to support language development in children with neurodevelopmental conditions. Research shows notable enhancements in expressive language, character recognition, morphological understanding, and phonological skills in preschool children with Attention-Deficit/Hyperactivity Disorder (ADHD) after targeted interventions (14), and significantly benefits vocabulary and listening comprehension skills in children with autism spectrum disorder (ASD) (15). During narrative processing in preschool children, left-hemisphere activation patterns were observed in regions associated with expressive language production, socioemotional integration, and working memory maintenance (16). Engaging in shared reading experiences is significantly linked to a lower incidence of emotional and behavioral problems (17, 18). Additionally, parent-child shared reading also enhances interaction between parents and children while promoting the psychological well-being of both (19, 20). Thus, shared reading is associated with better language skills, emotional control, and parent-child bonding, as well as fewer emotional and behavioral challenges.

A range of serious psychological conditions, including internalizing issues like anxiety and depression, neurodevelopmental disorders such as ADHD and autism, and externalizing behaviors like conduct problems, characterize children’s emotional and behavioral challenges, leading to significant functional impairments and adverse mental health outcomes (21). Research indicates that emotional and behavioral problems exhibit a high prevalence rate among preschool-aged children across diverse global populations, including in China (22–24). It is well known that the preschool period represents a critical developmental phase characterized by accelerated growth trajectories, multidimensional maturation processes, and remarkable neuroplasticity. This ontogenetic stage demonstrates distinctive developmental signatures across physiological, cognitive, affective, and socio-communicative domains, with particularly pronounced sensitivity to both endogenous (e.g., genetic, epigenetic) and exogenous (e.g., familial, socio-ecological) environmental determinants.

However, it is important to acknowledge that the relationship between the home environment and child developmental outcomes may be bidirectional rather than unidirectional. While extensive research has focused on how parents shape the home literacy environment, growing evidence indicates that children’s own characteristics, developmental status, and behavioral profiles can also significantly influence the nature and quality of their home environment. For instance, raising a child with autism spectrum disorder—characterized by severe deficits in social interaction and communication—has been associated with heightened maternal perceptions of burden, stress, and care complexity, which may substantially alter household routines, predictability, and overall environmental quality (25). Similarly, among school-aged children, maternal reports of parenting stress and psychological distress (including depression and anxiety) have been found to correlate significantly with child disruptive behaviors such as attention problems and aggression, suggesting that challenging child behaviors may actively contribute to a more chaotic and stressful home environment (26). These findings collectively support a reciprocal framework: while a disorganized or less enriching home environment may exacerbate children’s emotional and behavioral difficulties (27, 28), children’s pre-existing behavioral profiles or developmental challenges can also evoke specific parental responses and modifications in the home setting. In the context of shared reading specifically, this bidirectionality suggests that a child’s inattention, oppositional behavior, or lack of engagement in shared reading may lead parents to reduce structured literacy activities or adopt less responsive interaction styles, which in turn could further delay the child’s developmental outcomes. Therefore, any comprehensive understanding of the preschool home environment must consider the reciprocal influences between children’s behavioral functioning and the environmental inputs they receive.

Home literacy environment has been widely recognized in research as a beneficial factor for children’s linguistic and cognitive development (2). However, most prior studies have relied on smaller sample sizes, limiting their generalizability and ability to explore nuanced effects, such as threshold-dependent associations or variations across demographic subgroups (17, 18). Given the high prevalence of emotional and behavioral challenges among preschoolers globally, including in China (22–24), and the critical neuroplasticity of this developmental stage, large-scale empirical research is needed to clarify the relationship between shared reading and children’s socioemotional outcomes. The current study addresses this gap by employing stratified cluster sampling with a substantial sample of 21,366 parent-child pairs in western China, using standardized scales (StimQ-P and SDQ) to examine the association between shared reading and emotional/behavioral problems, as well as prosocial behaviors. By identifying potential thresholds and subgroup variations, this research aims to provide robust evidence regarding the associations between literacy practices and socioemotional resilience in preschoolers.

## Methods

### Research methodology and sample selection

This study was conducted in a major city in northern Shaanxi (western China), selected for its diverse administrative districts encompassing urban, suburban, and rural populations across the Loess Plateau, which reflect broader socioeconomic conditions in the northwestern region. Stratification variables included 13 administrative districts and counties, with clusters defined as individual public kindergartens. Random selection of kindergartens occurred within each stratum, and proportional allocation ensured sample sizes reflected district-level child populations. Given the negligible clustering effect (ICC < 0.001, see **Supplementary Table 1**), cluster-robust standard errors were not required for the primary analyses.

A total of 189 kindergartens were identified as recruitment sites, encompassing a diverse range of urban and rural areas. Children aged 3 to 6 years from junior, middle, and senior kindergarten classes, together with their parents, were invited to join the study. Children with known developmental disabilities or special needs were not systematically excluded from the study, as specific diagnostic information was not collected in the parent survey. Data collection occurred between February 28 and March 5, 2025. Written informed consent was secured from parents before participation, ensuring ethical standards and voluntary engagement. Data collection was carried out through a questionnaire distributed by the kindergartens. Each participating family was represented by one parent, who completed the survey on behalf of their child. Initially, 25,017 mother or father-child pairs participated in the survey, reflecting a robust response rate. Exclusions were primarily due to non-parent respondents (n=2,408; 9.6%, often rural grandparents), missing parent’s age (n=517; 2.1%), and missing child’s age (n=726; 2.9%). A comparison of available data showed no significant differences in district distribution between included and excluded samples (P>0.05), but potential selection bias cannot be fully excluded. After these exclusions, the final dataset comprised 21,366 valid samples, which were used for subsequent statistical analyses. The study was conducted according to the guidelines of the Declaration of Helsinki, and approval from the Ethics Committee of the Affiliated Children’s Hospital of Xi’an Jiaotong University was obtained (No.20250225-21).

### Variables

#### Emotional/behavioral problems

The official simplified Chinese version of the Strengths and Difficulties Questionnaire (SDQ) is a well-established instrument designed to evaluate the emotional and behavioral problems faced by children between the ages of 3 and 17 (29–31). This 25-item questionnaire evaluates five domains—emotional issues, conduct difficulties, hyperactivity, peer problems, and prosocial behavior—with each domain containing five items. Each item receives a score on a scale of three points (0-2), resulting in scores for each subdomain that vary from 0 to 10. A total difficulties score, obtained through the first four distinct subscales, ranges from 0 to 40, flags children at risk for emotional/behavioral problems when exceeding 14 points (30, 32). Specific items (7, 11, 14, 21, and 25) are scored in reverse. Prosocial behavior was dichotomized as ‘adequate’ (score ≥ 6) versus ‘deficit’ (score < 6) based on standard previous studies (30, 32). In this study, the total difficulties score and prosocial behavior score are utilized as dependent variables. The Cronbach’s alpha for the total difficulties score was 0.703. For the prosocial behavior subscale, Cronbach’s alpha was 0.826.

#### Shared reading

The shared reading was evaluated using the preschool version of StimQ (StimQ-P), a validated parent-report instrument designed for children between 36 and 72 months old (33). This measure, composed mainly of yes/no items, assesses four key areas: 1) Parental Verbal Responsivity, which focuses on conversational exchanges during routine activities; 2) Parental Involvement in Developmental Advance, evaluating educational interactions such as object naming and counting; 3) Reading, analyzing shared book-reading practices; and 4) Availability of learning materials, inventorying educational resources in the home. Researchers can examine either specific components or the full scale (33), though the current investigation focused exclusively on the read component. “Quantity (scored 0 to 9 points), Diversity of concept (scored 0 to 4 points), diversity of content (scored 0 to 4 points), and interactivity quality (scored 0 to 5 points) constitute the four reading dimensions assessed by this tool, with their scores combined to yield a total reading score of 0 to 22 points (3, 4), where elevated scores reflected more robust literacy environments. The Cronbach’s alpha of StimQ-P in the present study is 0.882.

#### Other factors

Based on previous literature and directed acyclic graph considerations, the following covariates were selected *a priori* as potential confounders and were adjusted for in all multivariable models.

Demographic and socioeconomic factors included: district (13 administrative divisions), kindergarten (adjusted as a covariate; null model analyses showed negligible between−kindergarten variance with intraclass correlation coefficient < 0.001, indicating no meaningful clustering effect; see **Supplementary Table 1**), household registration of children (urban vs. rural, reflecting the Chinese household registration system), child age (years, continuous), child gender (boys vs. girls), parental age (years, continuous), parental gender (male vs. female), education level (categorized as ≤ junior high school, high school diploma or junior college, or ≥ undergraduate degree), employment status (working vs. not working), annual family income (categorized as < 30,000 ¥, 30,000–100,000 ¥, or ≥ 100,000 ¥), marital status (married or living with partner vs. others), and number of children in the household (1, 2, or ≥ 3).

Lifestyle and behavioral factors included: average outdoor time (minutes per day, continuous), average screen time (minutes per day, continuous), smoking status (yes vs. no), and alcohol intake status (yes vs. no). Outdoor time and screen time were reported by parents as typical daily durations for the child. Parental smoking and alcohol use were assessed via self-report.

Parental mental health was assessed using the Center for Epidemiologic Studies Depression Scale (CES-D), a 20-item self-report scale measuring depressive symptoms in the general population. Total scores range from 0 to 60, with higher scores indicating greater depressive symptom severity. The CES-D has been validated in Chinese populations and demonstrated good internal consistency in this study (Cronbach’s α = 0.85).

These covariates were selected because they are known to influence both the home literacy environment (i.e., shared reading practices) and child socioemotional development, thereby potentially confounding the association between shared reading and emotional/behavioral outcomes. All covariates were measured at baseline via the parent-reported questionnaire.

### Statistical analysis

Continuous variables following a normal distribution were presented as means ± standard deviations, and categorical variables were presented as counts and proportions. Group comparisons were performed using one−way ANOVA for continuous variables and Pearson’s χ² test (or Fisher’s exact test when appropriate) for categorical variables. Missing data were handled using multiple imputation by chained equations (MICE) with 5 imputed datasets, and pooled regression estimates were calculated using Rubin’s rules. Multicollinearity among predictors was assessed using variance inflation factor (VIF), with VIF > 10 considered indicative of problematic collinearity; all VIF values were ≤ 2.4, indicating no significant multicollinearity. To assess whether the sampling design (children nested within kindergartens) required multilevel modeling, we fit null models with kindergarten as a random intercept. The intraclass correlation coefficients (ICC) were < 0.001 for both outcomes, indicating that less than 0.1% of the variance in outcomes was attributable to between−kindergarten differences. Therefore, standard regression models without random effects were used, and kindergarten was included as a fixed covariate in all adjusted models. Multivariable logistic regression was used to examine associations between shared reading (read scale score and its four subdomains) and binary outcomes (emotional/behavioral problems: yes/no; prosocial behavior adequate: yes/no). Three models were fitted: a non−adjusted model; Model I, adjusted for demographic and socioeconomic covariates (district, kindergarten, household registration, child age and gender, parental age and gender, education level, employment status, annual family income, marital status, number of children); and Model II, further adjusted for lifestyle factors (average outdoor time, average screen time, smoking status, alcohol intake status) and parental mental health (CES-D score). Results were presented as odds ratios (OR) with 95% confidence intervals (CIs). To examine potential nonlinear associations between shared reading score and child outcomes (total difficulties score and prosocial behavior score), we first used generalized additive models (GAMs) with penalized splines to visualize the dose−response relationship. Based on the observed nonlinear patterns, a two−piecewise linear regression model was then fitted to identify the optimal turning point (threshold, K) using a recursive algorithm. The 95% confidence interval for K was estimated using non−parametric bootstrap with 1,000 resamples, and a likelihood ratio test (LRT) comparing the two−piecewise model with a standard linear model was used to test the significance of the threshold effect. Stratified analyses were conducted to explore effect modifications by child age, child gender, parental age, parental gender, education level, annual family income, employment status, marital status, smoking status, and alcohol intake status, with interaction terms tested using the likelihood ratio test. Given the exploratory nature of the subgroup analyses, the Benjamini−Hochberg false discovery rate (FDR) method was applied to correct for multiple comparisons in the interaction tests. Several sensitivity analyses were conducted, including multivariable linear regression using continuous outcome scores, multiple imputation for missing data, and adjustment for kindergarten using cluster−robust standard errors, all of which yielded similar results. All statistical analyses were performed using R (version 4.2) and EmpowerStats, and a two−tailed P < 0.05 was considered statistically significant.

## Results

### Characteristics of the study participants

All VIF values were ≤ 2.4, indicating no significant multicollinearity (**Supplementary Tables 2**, **3**). Variables with missing values included non−parent respondent status (n = 2,408), parental age (n = 517), and child age (n = 726). Five imputed datasets were generated, and pooled regression estimates are reported (**Supplementary Tables 4**-**6**).

**Table 1** shows that among 21,366 preschoolers, 3,978 (18.6%) had emotional/behavioral problems (total difficulties score > 14). Compared with the non-problem group (n = 17,388), children with problems had less outdoor time (110.51 vs. 116.22 min) and more screen time (79.92 vs. 67.18 min) and were more likely to be boys (54.42% vs. 51.17%) and have rural registration (71.77% vs. 65.53%, all p < 0.001). Their parents had lower education (≤ junior high: 35.29% vs. 24.94%), lower income (< 30,000 ¥: 49.45% vs. 38.26%), and higher rates of smoking (18.68% vs. 14.01%) and alcohol use (21.07% vs. 15.36%, all p < 0.001). The problem group also had higher CES-D scores (15.25 vs. 10.27) and lower prosocial behavior scores (5.80 vs. 7.02, both p < 0.001). All shared reading components—total score, quantity, diversity of concepts and content, and interactivity quality—were significantly lower in the problem group (all p < 0.001).

**Table 1:** Descriptive characteristics of preschool children by total difficulties score in a western Chinese city.

Variables	Total (n=21366)	Non-Emotional/behavioral problems (Total difficulties score ≤ 14) (n = 17388)	Emotional/behavioral problems (Total difficulties score > 14) (n = 3978)	Standardize diff.	P-value
Child age (years)	4.82 ± 0.89	4.82 ± 0.88	4.80 ± 0.90	0.02 (-0.01, 0.05)	0.256
Child gender				0.07 (0.03, 0.10)	<0.001
Boys	11062 (51.77%)	8897 (51.17%)	2165 (54.42%)		
Girls	10304 (48.23%)	8491 (48.83%)	1813 (45.58%)		
Household registration of children				0.13 (0.10, 0.17)	<0.001
Urban	7116 (33.31%)	5993 (34.47%)	1123 (28.23%)		
Rural	14250 (66.69%)	11395 (65.53%)	2855 (71.77%)		
Average outdoor time	115.16 ± 61.20	116.22 ± 60.37	110.51 ± 64.52	0.09 (0.06, 0.13)	<0.001
Average screen time	69.55 ± 58.89	67.18 ± 57.54	79.92 ± 63.39	0.21 (0.18, 0.24)	<0.001
Parental age (years)	34.75 ± 4.56	34.81 ± 4.53	34.48 ± 4.68	0.07 (0.04, 0.11)	<0.001
Parental gender				0.14 (0.10, 0.17)	<0.001
Male	5108 (23.91%)	3961 (22.78%)	1147 (28.83%)		
Female	16258 (76.09%)	13427 (77.22%)	2831 (71.17%)		
Education level				0.25 (0.21, 0.28)	<0.001
≤ Junior high school	5741 (26.87%)	4337 (24.94%)	1404 (35.29%)		
High school diploma and junior college	9476 (44.35%)	7800 (44.86%)	1676 (42.13%)		
≥ Undergraduate degree	6149 (28.78%)	5251 (30.20%)	898 (22.57%)		
Employment status				0.12 (0.09, 0.16)	<0.001
Not working	5724 (26.79%)	4476 (25.74%)	1248 (31.37%)		
Working	15642 (73.21%)	12912 (74.26%)	2730 (68.63%)		
Annual family income, in thousands, ¥				0.24 (0.21, 0.28)	<0.001
< 30	8619 (40.34%)	6652 (38.26%)	1967 (49.45%)		
≥ 30 to 100	8619 (40.34%)	7168 (41.22%)	1451 (36.48%)		
≥ 100	4128 (19.32%)	3568 (20.52%)	560 (14.08%)		
Marital status				0.08 (0.04, 0.11)	<0.001
Married/Living with partner	20761 (97.17%)	16939 (97.42%)	3822 (96.08%)		
Others	605 (2.83%)	449 (2.58%)	156 (3.92%)		
Number of children			0.004	0.06 (0.02, 0.09)	
1	6148 (28.77%)	4981 (28.65%)	1167 (29.34%)		
2	12844 (60.11%)	10527 (60.54%)	2317 (58.25%)		
≥ 3	2374 (11.11%)	1880 (10.81%)	494 (12.42%)		
Smoking status				0.13 (0.09, 0.16)	<0.001
No	18187 (85.12%)	14952 (85.99%)	3235 (81.32%)		
Yes	3179 (14.88%)	2436 (14.01%)	743 (18.68%)		
Alcohol intake status				0.15 (0.11, 0.18)	<0.001
No	17858 (83.58%)	14718 (84.64%)	3140 (78.93%)		
Yes	3508 (16.42%)	2670 (15.36%)	838 (21.07%)		
CES-D	11.20 ± 6.62	10.27 ± 6.00	15.25 ± 7.60	0.73 (0.69, 0.76)	<0.001
Prosocial behavior	6.79 ± 2.14	7.02 ± 2.15	5.80 ± 1.78	0.62 (0.58, 0.65)	<0.001
Read scale	15.36 ± 3.42	15.55 ± 3.34	14.55 ± 3.65	0.29 (0.25, 0.32)	<0.001
Quantity	4.11 ± 2.13	4.22 ± 2.15	3.63 ± 1.95	0.29 (0.26, 0.32)	<0.001
Diversity of concepts	3.68 ± 0.63	3.68 ± 0.61	3.64 ± 0.70	0.07 (0.04, 0.11)	<0.001
Diversity of content	3.24 ± 1.01	3.26 ± 0.98	3.15 ± 1.12	0.11 (0.07, 0.14)	<0.001
Interactivity quality	4.34 ± 1.18	4.38 ± 1.12	4.14 ± 1.39	0.19 (0.16, 0.23)	<0.001

Continuous variables are presented as means ± standard deviations; categorical variables are presented as numbers (percentages). Standardized differences (Std. diff) with 95% confidence intervals and P-values were calculated for comparisons between children with emotional/behavioral problems (total difficulties score > 14) and those without (total difficulties score ≤ 14). CES-D, Center for Epidemiologic Studies Depression Scale. *P < 0.05.

### The association between shared reading and emotional/behavioral problems and prosocial behavior

**Table 2** displays the associations between shared reading components and both emotional/behavioral problems and prosocial behavior using logistic regression models. For emotional/behavioral problems, each one-point increase in the read scale score was associated with a reduction in the odds of problems in the non-adjusted model (OR = 0.92, 95% CI: 0.91–0.93, p < 0.0001), after adjusting for demographic and socioeconomic covariates (Model I: OR = 0.94, 95% CI: 0.93–0.95, p < 0.0001), and after additional adjustment for lifestyle and parental mental health factors (Model II: OR = 0.96, 95% CI: 0.95–0.97, p < 0.0001). Similarly, all subdomains of shared reading (quantity, diversity of concepts, diversity of content, and interactivity quality) showed significant associations in Model II, with ORs ranging from 0.92 to 0.96 (all p < 0.05). For prosocial behavior (adequate vs. inadequate), each one-point increase in the read scale score was associated with significantly higher odds of adequate prosocial behavior in Model II (OR = 1.09, 95% CI: 1.08–1.10, p < 0.0001). The subdomains also showed positive associations, with ORs ranging from 1.13 to 1.16 in the fully adjusted model (all p < 0.0001). These findings indicate that higher levels of shared reading are independently associated with fewer emotional/behavioral problems and better prosocial development.

**Table 2:** Associations between shared reading and emotional/behavioral problems and prosocial behavior in preschool children.

Exposure	Non-adjusted model	Adjusted model I	Adjusted model II
Emotional/behavioral problems			
Read scale	0.92 (0.91, 0.93) <0.0001	0.94 (0.93, 0.95) <0.0001	0.96 (0.95, 0.97) <0.0001
Quantity	0.87 (0.85, 0.88) <0.0001	0.90 (0.88, 0.91) <0.0001	0.92 (0.90, 0.93) <0.0001
Diversity of concepts	0.89 (0.84, 0.94) <0.0001	0.88 (0.83, 0.93) <0.0001	0.92 (0.88, 0.98) 0.0058
Diversity of content	0.90 (0.87, 0.93) <0.0001	0.93 (0.90, 0.96) <0.0001	0.96 (0.93, 1.00) 0.0433
Interactivity quality	0.85 (0.83, 0.88) <0.0001	0.88 (0.85, 0.90) <0.0001	0.93 (0.90, 0.96) <0.0001
Prosocial behavior adequate			
Read scale	1.11 (1.10, 1.12) <0.0001	1.10 (1.09, 1.11) <0.0001	1.09 (1.08, 1.10) <0.0001
Quantity	1.16 (1.15, 1.18) <0.0001	1.16 (1.14, 1.17) <0.0001	1.13 (1.11, 1.14) <0.0001
Diversity of concepts	1.17 (1.12, 1.23) <0.0001	1.13 (1.08, 1.18) <0.0001	1.16 (1.11, 1.22) <0.0001
Diversity of content	1.19 (1.16, 1.22) <0.0001	1.16 (1.13, 1.19) <0.0001	1.15 (1.12, 1.19) <0.0001
Interactivity quality	1.20 (1.17, 1.23) <0.0001	1.19 (1.16, 1.22) <0.0001	1.16 (1.13, 1.18) <0.0001

Data are presented as odds ratios (OR) with 95% confidence intervals (CIs) and P-values. Odds ratios are per one-point increase in the read scale or subscale score. Non-adjusted model adjusted for none. Adjusted Model I adjusted for district; kindergarten; household registration of children; child age; child gender; parental age; parental gender; education level; employment status; annual family income; marital status; number of children. Adjusted Model II adjusted for all variables in Model I plus average outdoor time; average screen time; CES-D score; smoking status; alcohol intake status.

Sensitivity analyses using multiple linear regression confirmed the primary findings. For emotional/behavioral problems (continuous total difficulties score), each one−point increase in the read scale was associated with a decrease of 0.28 in the non−adjusted model (β = -0.28, 95% CI: -0.30 to -0.26, p < 0.0001), and a decrease of 0.17 after full adjustment (β = -0.17, 95% CI: -0.19 to -0.16, p < 0.0001). All subdomains also showed significant negative associations. For prosocial behavior (continuous score), the read scale was positively associated in the non−adjusted model (β = 0.13, 95% CI: 0.12–0.14, p < 0.0001) and remained significant after full adjustment (β = 0.11, 95% CI: 0.10–0.12, p < 0.0001). These continuous analyses reinforce the robustness of the relationships observed in the binary outcome models (**Supplementary Table 7**).

**Supplementary Table 1** shows that the between−kindergarten variances for emotional/behavioral problems (4.64×10^-^¹^4^) and prosocial behavior (0.00) were near zero, with ICCs <0.001 and 0.000, respectively. Both models had singular fit, indicating no meaningful clustering by kindergarten. Therefore, standard regression models without random effects were appropriate for the primary analyses.

### Threshold effects of shared reading on emotional/behavioral problems and prosocial behavior

To explore potential nonlinear relationships, a two-piecewise linear regression model was fitted, adjusting for all covariates. This analysis identified a significant threshold effect for both total difficulties and prosocial behavior scores (P for likelihood ratio test < 0.001 for both outcomes; **Table 3**).

**Table 3:** Threshold effect analysis of shared reading on total difficulties and prosocial behavior in preschool children using a two-piecewise linear model.

Outcome	Turning point K	CI lower	CI upper	Beta below K	Beta above K	P for LRT
Total difficulties score	17.9	17	19	-0.13	-0.33	<0.001
Prosocial behavior score	15	14	17	0.08	0.13	<0.001

K represents the optimal turning point (threshold) of shared reading score, with 95% confidence intervals (CIs) estimated via bootstrap. β values represent the change in outcome score per one-point increase in shared reading score below and above the turning point. P for LRT (likelihood ratio test) tests the significance of the threshold effect (non-linearity). All models were fully adjusted for district; kindergarten; household registration of children; child age; child gender; parental age; parental gender; education level; employment status; annual family income; marital status; number of children; average outdoor time; average screen time; CES-D score; smoking status; alcohol intake status.

**Figure 1:**
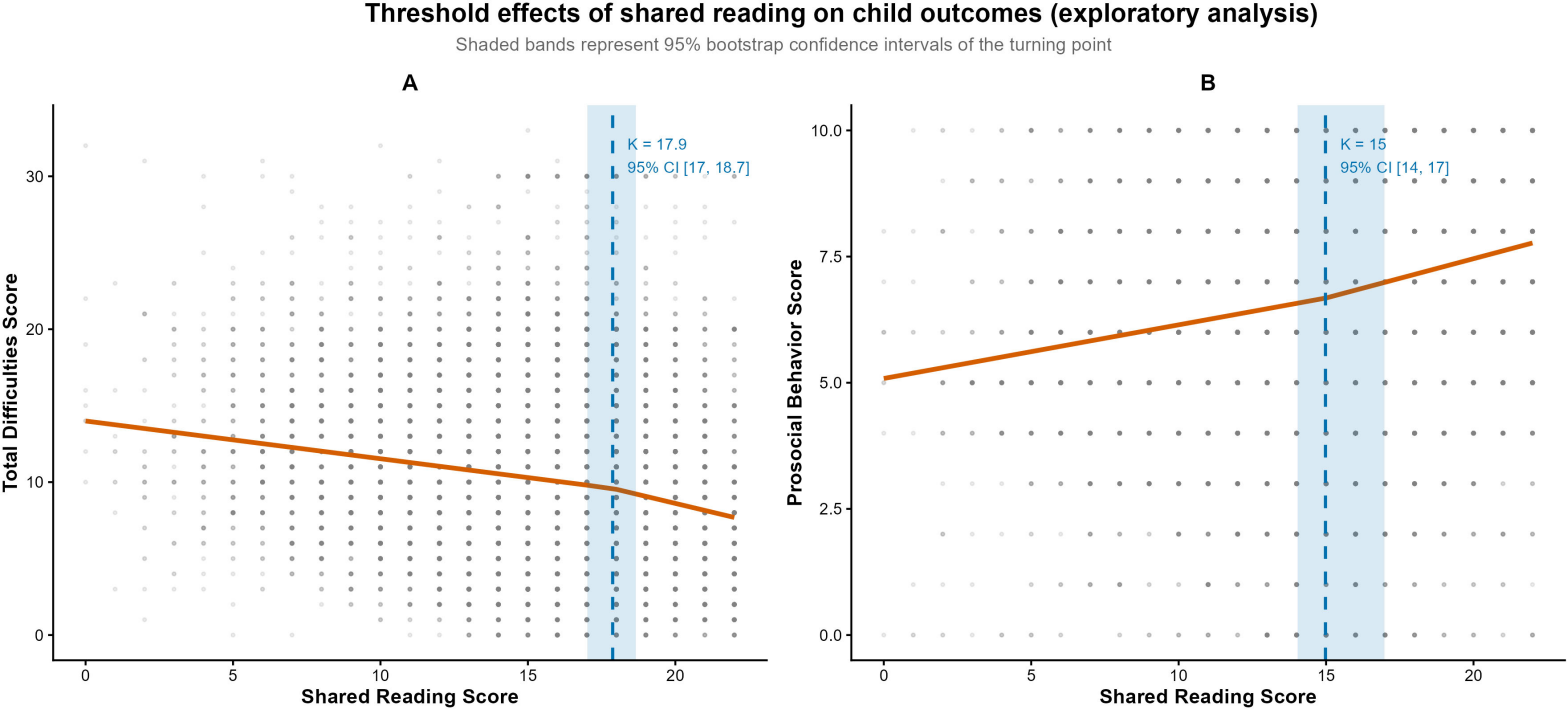
Threshold effects of shared reading on emotional/behavioral problems and prosocial behavior. **(A)** Association between shared reading score and total difficulties score. **(B)** Association between shared reading score and prosocial behavior score. Solid lines represent the predicted relationship between shared reading and each outcome based on a fully adjusted two-piecewise linear model (covariates adjusted are detailed in the note of **Table 3**). Vertical dashed lines indicate the optimal turning point (K) estimated by the model. Shaded bands represent the 95% bootstrap confidence intervals (CIs) for the turning point. In **(A)**, the turning point was estimated at K = 17.9 (95% CI: 17.0, 18.7), suggesting a steeper reduction in total difficulties scores above this threshold. In **(B)**, the turning point was K = 15.0 (95% CI: 14.0, 17.0), indicating a greater increase in prosocial behavior scores above this threshold.

For the total difficulties score, the turning point (K) was estimated at 17.87 (95% CI: 17.02, 18.66). Below this threshold, each one-point increase in the shared reading score was associated with a minimal change in the total difficulties score (β = -0.13). However, above the threshold, the association strengthened substantially, with each one-point increase in shared reading corresponding to a reduction of -0.33 points in the total difficulties score. This pattern was visually confirmed in **Figure 1A**, which illustrates a shallow slope before the turning point (indicated by the vertical line at K = 17.9) and a steeper negative slope thereafter. The shaded bands representing the 95% bootstrap confidence interval for the turning point further support the location of this threshold.

For the prosocial behavior score, the turning point was identified at 14.98 (95% CI: 14.03, 16.98; **Table 3**). The positive association between shared reading and prosocial behavior was also nonlinear. Below the threshold, the benefit was modest (β = 0.08 per one-point increase), whereas above the threshold, the benefit increased to β = 0.13 per one-point increase. This nonlinear pattern is depicted in **Figure 1B**, where the slope of the line becomes noticeably steeper after the turning point at K = 15. The 95% CI for this turning point (14, 17) is also indicated, showing a consistent range across both the table and figure.

### Stratified analysis between shared reading emotional/behavioral problems and prosocial behavior

**Figure 2:**
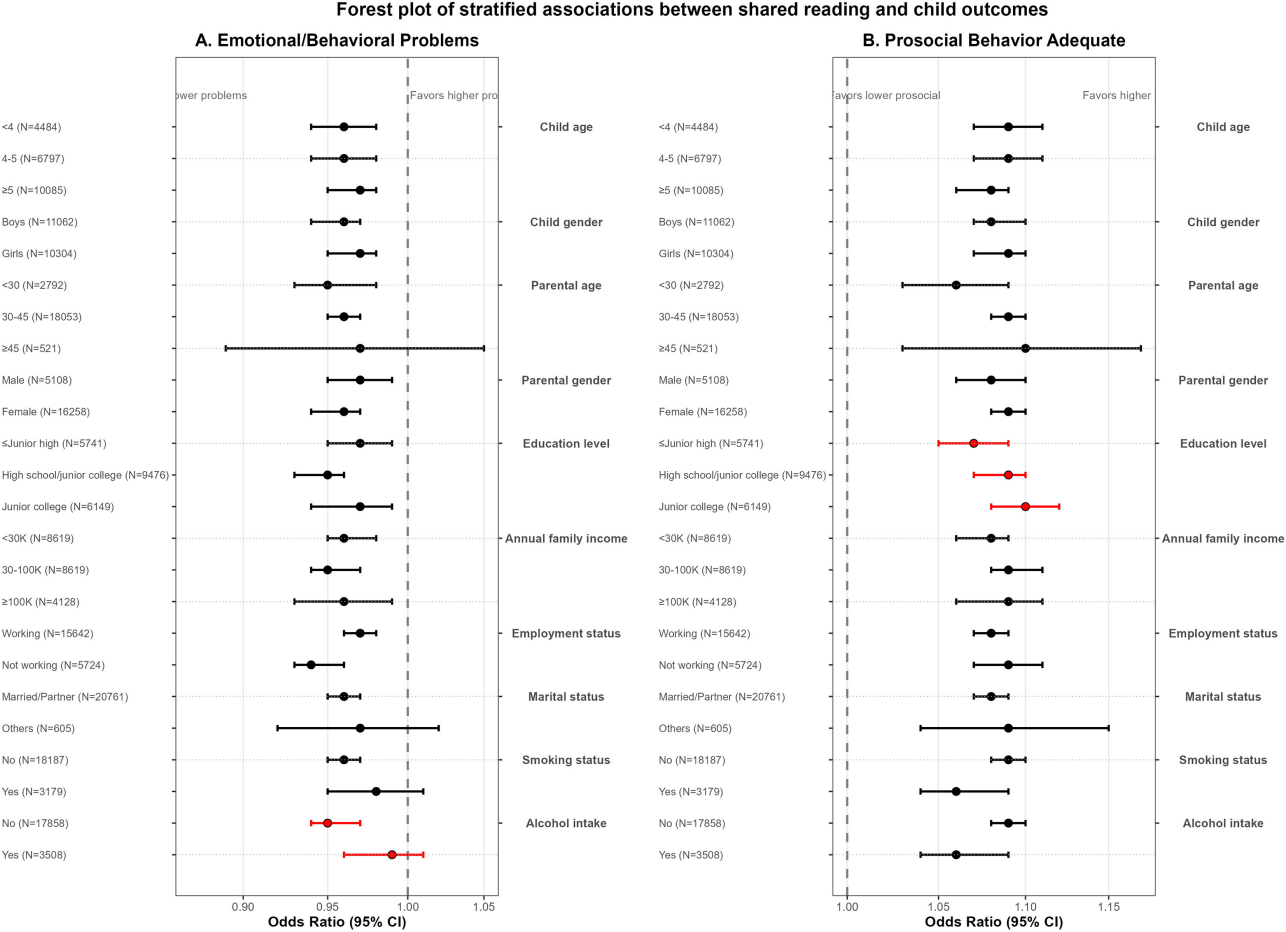
Forest plot of stratified associations between shared reading and emotional/behavioral problems and prosocial behavior. **(A)** Associations between shared reading score and emotional/behavioral problems across subgroups. **(B)** Associations between shared reading score and adequate prosocial behavior across subgroups. Odds ratios (ORs) with 95% confidence intervals (CIs) are presented for each subgroup, representing the change in the odds of the outcome per one-point increase in shared reading score. The vertical dashed line at OR = 1.0 indicates the null effect. For emotional/behavioral problems **(A)**, ORs < 1.0 favor lower risk. For prosocial behavior adequate **(B)**, ORs > 1.0 favor higher likelihood of adequate prosocial behavior. All models were fully adjusted for all covariates listed in **Table 3**, except for the stratification variable itself. P for interaction values are reported in **Table 4**.

Stratified analyses were exploratory and examined whether the associations between shared reading and child outcomes varied across subgroups (**Table 4**, **Figure 2**). Higher shared reading scores were significantly associated with lower odds of emotional/behavioral problems (OR range: 0.94–0.99) and higher odds of adequate prosocial behavior (OR range: 1.06–1.10) across most subgroups, with the majority of associations remaining significant (all P < 0.05). Interaction tests were performed for 10 stratifiers. To account for multiple testing, FDR correction was applied. After correction, only parental alcohol consumption status showed a significant interaction with shared reading for emotional/behavioral problems (FDR−adjusted P = 0.042). No other interactions, including parental gender, education level, or smoking status, remained significant after FDR correction (all adjusted P > 0.05). For prosocial behavior, no significant interactions were observed after correction. These results suggest that the beneficial associations of shared reading are largely consistent across subgroups, with limited evidence of effect modification.

**Table 4:** Stratified analysis of associations between shared reading and emotional/behavioral problems and prosocial behavior in preschool children.

Variable	Subgroup	N	Emotional/behavioral problems OR (95% CI) P value	Prosocial behavior adequate OR (95% CI) P value	P for interaction (EBP)	P for interaction (Prosocial)
Child age	<4	4,484	0.96 (0.94-0.98) <0.001	1.09 (1.07-1.11) <0.001	0.245	0.345
	4-5	6,797	0.96 (0.94-0.98) <0.001	1.09 (1.07-1.11) <0.001		
	≥ 5	10,085	0.97 (0.95-0.98) <0.001	1.08 (1.06-1.09) <0.001		
Child gender	Boys	11,062	0.96 (0.94-0.97) <0.001	1.08 (1.07-1.10) <0.001	0.156	0.267
	Girls	10,304	0.97 (0.95-0.98) <0.001	1.09 (1.07-1.10) <0.001		
Parental age	<30	2,792	0.95 (0.93-0.98) <0.001	1.06 (1.03-1.09) <0.001	0.089	0.178
	30-45	18,053	0.96 (0.95-0.97) <0.001	1.09 (1.08-1.10) <0.001		
	≥ 45	521	0.97 (0.89-1.05) 0.385	1.10 (1.03-1.17) 0.002		
Parental gender	Male	5,108	0.97 (0.95-0.99) 0.010	1.08 (1.06-1.10) <0.001	0.167	0.234
	Female	16,258	0.96 (0.94-0.97) <0.001	1.09 (1.08-1.10) <0.001		
Education level	≤ Junior high school	5,741	0.97 (0.95-0.99) 0.001	1.07 (1.05-1.09) <0.001	0.187	0.045
	High school	9,476	0.95 (0.93-0.96) <0.001	1.09 (1.07-1.10) <0.001		
	Junior college	6,149	0.97 (0.94-0.99) 0.003	1.10 (1.08-1.12) <0.001		
Annual family income	<30K	8,619	0.96 (0.95-0.98) <0.001	1.08 (1.06-1.09) <0.001	0.234	0.345
	30-100K	8,619	0.95 (0.94-0.97) <0.001	1.09 (1.08-1.11) <0.001		
	≥100K	4,128	0.96 (0.93-0.99) 0.010	1.09 (1.06-1.11) <0.001		
Employment status	Working	15,642	0.97 (0.96-0.98) <0.001	1.08 (1.07-1.09) <0.001	0.678	0.567
	Not working	5,724	0.94 (0.93-0.96) <0.001	1.09 (1.07-1.11) <0.001		
Marital status	Married/Living with partner	20,761	0.96 (0.95-0.97) <0.001	1.08 (1.07-1.09) <0.001	0.456	0.345
	Others	605	0.97 (0.92-1.02) 0.249	1.09 (1.04-1.15) <0.001		
Smoking status	No	18,187	0.96 (0.95-0.97) <0.001	1.09 (1.08-1.10) <0.001	0.123	0.234
	Yes	3,179	0.98 (0.95-1.01) 0.130	1.06 (1.04-1.09) <0.001		
Alcohol intake status	No	17,858	0.95 (0.94-0.97) <0.001	1.09 (1.08-1.10) <0.001	0.042	0.567
	Yes	3,508	0.99 (0.96-1.01) 0.354	1.06 (1.04-1.09) <0.001		

Data are presented as odds ratios (OR) with 95% confidence intervals (CIs) and P-values. Odds ratios are per one-point increase in the read scale score. P for interaction values were calculated to test for effect modification by each subgroup variable. All models were adjusted for district; kindergarten; household registration of children; child age; child gender; parental age; parental gender; education level; employment status; annual family income; marital status; number of children; average outdoor time; average screen time; CES-D score; smoking status; alcohol intake status, except for the stratification variable itself. EBP, emotional/behavioral problems.

## Discussion

This large cross-sectional study of over 21,000 preschool children in western China documented an association between richer shared reading practices and a lower likelihood of emotional and behavioral problems, as well as a higher likelihood of adequate prosocial behavior. These associations persisted after accounting for a comprehensive set of demographic, socioeconomic, lifestyle, and parental mental health covariates. The consistency of the findings across multiple sensitivity analyses—including linear regression with continuous outcomes, multiple imputation for missing data, and adjustment for kindergarten-level clustering—supports the robustness of the observed patterns. Importantly, the relationship was not uniform across the range of shared reading scores. A threshold pattern emerged, such that the strength of the association between shared reading and both total difficulties and prosocial behavior appeared to increase markedly above certain score levels. Below those estimated thresholds, the associations were modest, whereas above them, each additional point on the read scale was associated with a substantially greater difference in difficulties (lower scores) and in prosocial behavior (higher scores). This pattern suggests the possibility that shared reading may need to reach a certain level of richness—in terms of quantity, diversity of concepts and content, and interactivity quality—before socioemotional differences become more pronounced. To our knowledge, this is among the first studies to document such non-linear, threshold-pattern associations in the context of shared reading and preschool mental health using a large community-based sample.

These findings align with and extend prior research. Previous smaller-scale studies have reported inverse associations between shared reading and child emotional or behavioral problems, as well as positive associations with social competence (34, 35). The present study adds precision and generalizability to those observations by employing a validated multidimensional measure of shared reading (the StimQ-P read scale) rather than single-item indicators of reading frequency (36). This distinction is relevant because shared reading is not a unidimensional activity; it encompasses how often families read, the range of concepts and content covered, and the quality of interactive dialogue during reading (37, 38). The results suggest that all four components—quantity, concept diversity, content diversity, and interactivity quality—showed independent associations with child outcomes, consistent with the theoretical framework that multiple dimensions of the home literacy environment collectively relate to child development (39). The finding that threshold patterns were evident for the total read scale but not systematically for individual subdomains further implies that it may be the accumulation of multiple enriching features of shared reading, rather than any single feature alone, that is associated with socioemotional differences.

Several hypothetical mechanisms have been proposed in the literature to explain why shared reading might be associated with child socioemotional outcomes, though these remain speculative and cannot be tested for causality in the present cross-sectional design. One hypothesis is that shared reading provides a structured, predictable, and emotionally contained context in which children may learn to label feelings, anticipate narrative resolutions, and practice turn-taking in dialogue (34). During shared reading, caregivers naturally model discussion of characters’ emotions and conflicts, which could hypothetically support children’s ability to recognize and manage emotional states (40). Another hypothesis is that shared reading exposes children to narratives that reward cooperation, empathy, and helping, potentially contributing to the internalization of social norms (41). A third hypothesis involves the parent–child relationship: shared reading has been associated with more positive parent–child interactions in previous research (37), and longitudinal work has shown that engaging in shared reading at younger ages is associated with less harsh parenting at later ages (42). Given that harsh parenting has been associated with unfavorable child emotional and behavioral outcomes (43), shared reading could hypothetically relate to child outcomes partly through its associations with parenting practices. A fourth hypothesis concerns language development: the linguistic and cognitive stimulation provided by shared reading has been linked to vocabulary growth and expressive language skills (44), and early vocabulary development has in turn been positively correlated with early social-emotional and behavioral development (45). Shared reading may therefore hypothetically relate to fewer behavioral difficulties through pathways involving enhanced communication skills. It is also plausible that these hypothesized mechanisms operate in combination and may differ across child age, gender, or family context. However, the subgroup analyses in the present study did not provide strong evidence of effect modification after correction for multiple testing, suggesting that the observed associations may be broadly consistent across diverse family circumstances. Nevertheless, the stratified analyses were exploratory, and the study was not designed with sufficient power specifically for interaction detection.

### Limitations

Several limitations warrant consideration. First, excluding non-parent respondents—many of whom were rural grandparents—may reduce sample representativeness. Although a sensitivity analysis including these respondents yielded similar results, differences in caregiving contexts or reporting styles cannot be ruled out. Future studies should oversample non-parental caregivers.

Second, both the exposure (shared reading practices) and outcomes (emotional/behavioral problems and prosocial behavior) were reported by the same parent at the same time point, introducing potential common-method bias. Parents who are more engaged, more educated, or prone to socially desirable responding might simultaneously report richer literacy practices and fewer behavioral difficulties, thereby inflating the observed associations. Although we adjusted for a wide range of socioeconomic and parental psychological variables (e.g., education, income, CES-D score), residual bias cannot be fully ruled out. Moreover, we acknowledge the potential for bidirectional relationships: children’s emotional or behavioral difficulties may reduce parental engagement in literacy activities. Our cross-sectional design cannot determine whether richer shared reading precedes better outcomes or whether children with fewer behavioral challenges elicit more positive parental interactions.

Additionally, the StimQ-P is a parent-reported measure; therefore, future studies could incorporate direct observational methods to validate the quality of parent-child reading interactions. To address these limitations, future studies should also use teacher-reported SDQ, multi-informant assessments, or longitudinal designs to reduce shared-method variance and clarify temporal relationships. Finally, the threshold analysis was exploratory, and the reported turning points may be specific to this sample. Overfitting is a concern, and the confidence intervals around the turning points were wide, indicating uncertainty in the exact threshold location.

## Conclusion

In this cross-sectional study of 21,366 preschool children in western China, higher levels of shared reading—encompassing greater quantity, broader diversity of concepts and content, and higher interactivity quality—were associated with lower odds of emotional and behavioral problems and higher odds of adequate prosocial behavior. The associations followed a threshold pattern: the differences in both total difficulties and prosocial behavior became more pronounced above estimated read scale scores of approximately 18 and 15, respectively. These associations were robust to adjustment for a wide range of demographic, socioeconomic, lifestyle, and parental mental health factors, and were largely consistent across child age, child gender, parental age, parental gender, education level, income, and employment status. However, given the cross-sectional design, no conclusions about directionality or causality can be drawn. The findings are hypothesis-generating and suggest that future longitudinal research should examine temporal patterns between shared reading and child socioemotional outcomes, and that experimental studies may be warranted to test whether enriching shared reading practices leads to subsequent improvements in preschool mental health. Until such evidence is available, efforts to support shared reading should be viewed as a potentially promising but unproven component of broader early childhood promotion strategies.

## Data Availability

The original contributions presented in the study are included in the article/**Supplementary Material**. Further inquiries can be directed to the corresponding author.
