# Supplementary material for: Shared reading is associated with fewer emotional/behavioral problems and better prosocial behavior in preschool children: a cross-sectional study in western China
Source: Front Psychiatry. 2026 Jun 24;17:1858077. doi: 10.3389/fpsyt.2026.1858077 (PMC13341906; doi:10.3389/fpsyt.2026.1858077)
Supplement: Supplementary file 1 [file Table1.docx]

Supplementary Table 1. Variance components and intraclass correlation coefficients (ICC) from null models with kindergarten as random intercept

| **Outcome** | **Between‑kindergarten variance** | **Within‑kindergarten variance** | **ICC** | **Model status** |
| --- | --- | --- | --- | --- |
| Emotional/behavioral problems | 4.64 × 10⁻¹⁴ | 0.1515 | < 0.001 | Singular fit |
| Prosocial behavior | 0.00 | 0.2500 | 0.000 | Singular fit |

ICC = between‑kindergarten variance / (between‑kindergarten variance + within‑kindergarten variance). For all three outcomes, the between‑kindergarten variance was essentially zero, indicating no meaningful clustering by kindergarten. Therefore, standard regression models (without random effects) are appropriate for the primary analyses.

Supplementary Table 2 Multicollinearity diagnostics for the regression model of emotional/behavioral problems

| **Emotional/behavioral problems** | **VIF** | **VIF** | **VIF** | **VIF** | **VIF** |
| --- | --- | --- | --- | --- | --- |
| **Read scale** | 1.1 |  |  |  |  |
| Quantity |  | 1.1 |  |  |  |
| Diversity of concepts |  |  | 1 |  |  |
| Diversity of content |  |  |  | 1.1 |  |
| Interactivity quality |  |  |  |  | 1.1 |
| **Kindergarten** | 1 | 1 | 1 | 1 | 1.1 |
| **Household registration of children** | 1.3 | 1.3 | 1.3 | 1.3 | 1 |
| **Child age** | 1 | 1 | 1.1 | 1 | 1.3 |
| **Child gender** | 1 | 1 | 1 | 1 | 1 |
| **Average outdoor time** | 1.1 | 1.1 | 1.1 | 1.1 | 1 |
| **Average screen time** | 1.2 | 1.2 | 1.1 | 1.2 | 1.1 |
| **Age** | 1.2 | 1.2 | 1.2 | 1.2 | 1.2 |
| **Gender** | 2.4 | 2.4 | 2.4 | 2.4 | 1.2 |
| **Education level** | 1.5 | 1.5 | 1.5 | 1.5 | 2.4 |
| **Employment status** | 1.3 | 1.3 | 1.3 | 1.4 | 1.5 |
| **Annual family income** | 1.1 | 1.2 | 1.1 | 1.1 | 1.4 |
| **Smoke status** | 2.3 | 2.3 | 2.3 | 2.3 | 1.1 |
| **Alcohol intake status** | 1.8 | 1.8 | 1.8 | 1.8 | 2.3 |
| **Marital status** | 1 | 1 | 1 | 1 | 1.8 |
| **CES-D** | 1.1 | 1.1 | 1.1 | 1.1 | 1 |
| **Number of children** | 1.2 | 1.2 | 1.2 | 1.2 | 1.1 |

Supplementary Table 3 Multicollinearity diagnostics for the regression model of prosocial behavior

| **Prosocial behavior** **adequate** | **VIF** | **VIF** | **VIF** | **VIF** | **VIF** |
| --- | --- | --- | --- | --- | --- |
| **Read scale** | 1.1 |  |  |  |  |
| Quantity |  | 1.1 |  |  |  |
| Diversity of concepts |  |  | 1 |  |  |
| Diversity of content |  |  |  | 1.1 |  |
| Interactivity quality |  |  |  |  | 1.1 |
| **Kindergarten** | 1 | 1 | 1 | 1 | 1 |
| **Household registration of children** | 1.3 | 1.3 | 1.3 | 1.3 | 1.3 |
| **Child age** | 1 | 1 | 1.1 | 1 | 1 |
| **Child gender** | 1 | 1 | 1 | 1 | 1 |
| **Average outdoor time** | 1.1 | 1.1 | 1.1 | 1.1 | 1.1 |
| **Average screen time** | 1.2 | 1.2 | 1.1 | 1.2 | 1.2 |
| **Age** | 1.2 | 1.2 | 1.2 | 1.2 | 1.2 |
| **Gender** | 2.4 | 2.4 | 2.4 | 2.4 | 2.4 |
| **Education level** | 1.5 | 1.5 | 1.5 | 1.5 | 1.5 |
| **Employment status** | 1.3 | 1.3 | 1.3 | 1.4 | 1.4 |
| **Annual family income** | 1.1 | 1.2 | 1.1 | 1.1 | 1.1 |
| **Smoke status** | 2.3 | 2.3 | 2.3 | 2.3 | 2.3 |
| **Alcohol intake status** | 1.8 | 1.8 | 1.8 | 1.8 | 1.8 |
| **Marital status** | 1 | 1 | 1 | 1 | 1 |
| **CES-D** | 1.1 | 1.1 | 1.1 | 1.1 | 1.1 |
| **Number of children** | 1.2 | 1.2 | 1.2 | 1.2 | 1.2 |

Supplemental Table 4 Distribution of missing variables.

| **Characteristic** | **Missing number** |
| --- | --- |
| Non-parent respondents | 2408 |
| Parent's age | 517 |
| Child's age | 726 |

Supplemental Table 5 Five sets of regression data after interpolation for missing data on non-parent respondents, parent's age and child's age.

| **Exposure** | **Non-adjusted Model** | **Non-adjusted Model** | **Non-adjusted Model** | **Non-adjusted Model** | **Non-adjusted Model** | **Adjusted Model** | **Adjusted Model** | **Adjusted Model** | **Adjusted Model** | **Adjusted Model** |
| --- | --- | --- | --- | --- | --- | --- | --- | --- | --- | --- |
| **Emotional/behavioral problems** |  |  |  |  |  |  |  |  |  |  |
| **Read scale** | 0.92 (0.91, 0.93) <0.0001 | 0.92 (0.91, 0.93) <0.0001 | 0.92 (0.91, 0.93) <0.0001 | 0.92 (0.91, 0.93) <0.0001 | 0.92 (0.91, 0.93) <0.0001 | 0.97 (0.96, 0.98) <0.0001 | 0.97 (0.96, 0.98) <0.0001 | 0.97 (0.96, 0.98) <0.0001 | 0.97 (0.96, 0.98) <0.0001 | 0.97 (0.96, 0.98) <0.0001 |
| Quantity | 0.87 (0.86, 0.89) <0.0001 | 0.87 (0.86, 0.89) <0.0001 | 0.87 (0.86, 0.89) <0.0001 | 0.87 (0.86, 0.89) <0.0001 | 0.87 (0.86, 0.89) <0.0001 | 0.94 (0.92, 0.96) <0.0001 | 0.94 (0.92, 0.96) <0.0001 | 0.94 (0.92, 0.96) <0.0001 | 0.94 (0.92, 0.96) <0.0001 | 0.94 (0.92, 0.96) <0.0001 |
| Diversity of concepts | 0.87 (0.83, 0.91) <0.0001 | 0.87 (0.83, 0.91) <0.0001 | 0.87 (0.83, 0.91) <0.0001 | 0.87 (0.83, 0.91) <0.0001 | 0.87 (0.83, 0.91) <0.0001 | 0.94 (0.90, 0.99) 0.0236 | 0.94 (0.90, 0.99) 0.0228 | 0.94 (0.90, 0.99) 0.0233 | 0.94 (0.90, 0.99) 0.0237 | 0.94 (0.90, 0.99) 0.0236 |
| Diversity of content | 0.90 (0.87, 0.92) <0.0001 | 0.90 (0.87, 0.92) <0.0001 | 0.90 (0.87, 0.92) <0.0001 | 0.90 (0.87, 0.92) <0.0001 | 0.90 (0.87, 0.92) <0.0001 | 0.95 (0.90, 0.99) <0.0001 | 0.94 (0.90, 0.99) <0.0001 | 0.96 (0.90, 0.98) <0.0001 | 0.93 (0.90, 0.96) <0.0001 | 0.94 (0.90, 0.96) <0.0001 |
| Interactivity quality | 0.85 (0.83, 0.87) <0.0001 | 0.85 (0.83, 0.87) <0.0001 | 0.85 (0.83, 0.87) <0.0001 | 0.85 (0.83, 0.87) <0.0001 | 0.85 (0.83, 0.87) <0.0001 | 0.94 (0.92, 0.97) <0.0001 | 0.94 (0.92, 0.97) <0.0001 | 0.94 (0.92, 0.97) <0.0001 | 0.94 (0.92, 0.97) <0.0001 | 0.94 (0.92, 0.97) <0.0001 |
| **Prosocial behavior adequate** |  |  |  |  |  |  |  |  |  |  |
| **Read scale** | 1.11 (1.10, 1.12) <0.0001 | 1.11 (1.10, 1.12) <0.0001 | 1.11 (1.10, 1.12) <0.0001 | 1.11 (1.10, 1.12) <0.0001 | 1.11 (1.10, 1.12) <0.0001 | 1.08 (1.07, 1.09) <0.0001 | 1.08 (1.07, 1.09) <0.0001 | 1.08 (1.07, 1.09) <0.0001 | 1.08 (1.07, 1.09) <0.0001 | 1.08 (1.07, 1.09) <0.0001 |
| Quantity | 1.17 (1.15, 1.18) <0.0001 | 1.17 (1.15, 1.18) <0.0001 | 1.17 (1.15, 1.18) <0.0001 | 1.17 (1.15, 1.18) <0.0001 | 1.17 (1.15, 1.18) <0.0001 | 1.12 (1.11, 1.14) <0.0001 | 1.12 (1.11, 1.14) <0.0001 | 1.12 (1.11, 1.14) <0.0001 | 1.12 (1.11, 1.14) <0.0001 | 1.12 (1.11, 1.14) <0.0001 |
| Diversity of concepts | 1.17 (1.12, 1.21) <0.0001 | 1.17 (1.12, 1.21) <0.0001 | 1.17 (1.12, 1.21) <0.0001 | 1.17 (1.12, 1.21) <0.0001 | 1.17 (1.12, 1.21) <0.0001 | 1.08 (1.03, 1.12) 0.0004 | 1.08 (1.03, 1.12) 0.0004 | 1.08 (1.03, 1.12) 0.0004 | 1.08 (1.03, 1.12) 0.0004 | 1.08 (1.03, 1.12) 0.0004 |
| Diversity of content | 1.19 (1.16, 1.22) <0.0001 | 1.19 (1.16, 1.22) <0.0001 | 1.19 (1.16, 1.22) <0.0001 | 1.19 (1.16, 1.22) <0.0001 | 1.19 (1.16, 1.22) <0.0001 | 1.11 (1.08, 1.14) <0.0001 | 1.11 (1.08, 1.14) <0.0001 | 1.11 (1.08, 1.14) <0.0001 | 1.11 (1.08, 1.14) <0.0001 | 1.11 (1.08, 1.14) <0.0001 |
| Interactivity quality | 1.19 (1.17, 1.22) <0.0001 | 1.19 (1.17, 1.22) <0.0001 | 1.19 (1.17, 1.22) <0.0001 | 1.19 (1.17, 1.22) <0.0001 | 1.19 (1.17, 1.22) <0.0001 | 1.13 (1.11, 1.16) <0.0001 | 1.13 (1.11, 1.16) <0.0001 | 1.13 (1.11, 1.16) <0.0001 | 1.13 (1.11, 1.16) <0.0001 | 1.13 (1.11, 1.16) <0.0001 |

The data is represented by OR (95% CI) P value, OR per 1-point increase in read scale score.

Non-adjusted model adjust for: None

Adjusted model adjust for: district; kindergarten; household registration of children; child age; child gender; parental age; parental gender; education level; employment status; annual family income; marital status; number of children; average outdoor time; average screen time; CES-D; smoking status; alcohol intake status

Supplementary Table 6 Associations between shared reading and emotional/behavioral problems and prosocial behavior in preschool children after imputing the baseline missing values

| **Exposure** | **Non-adjusted Model** | **Adjusted Model** |
| --- | --- | --- |
| **Emotional/behavioral problems** |  |  |
| **Read scale** | 0.92 (0.91, 0.93) <0.0001 | 0.97 (0.96, 0.98) <0.0001 |
| Quantity | 0.87 (0.86, 0.89) <0.0001 | 0.94 (0.92, 0.96) <0.0001 |
| Diversity of concepts | 0.87 (0.83, 0.91) <0.0001 | 0.94 (0.90, 0.99) 0.0109 |
| Diversity of content | 0.90 (0.88, 0.93) <0.0001 | 0.94 (0.90, 0.99) 0.0171 |
| Interactivity quality | 0.85 (0.83, 0.87) <0.0001 | 0.94 (0.92, 0.97) <0.0001 |
| **Prosocial behavior adequate** |  |  |
| **Read scale** | 1.11 (1.10, 1.12) <0.0001 | 1.08 (1.07, 1.09) <0.0001 |
| Quantity | 1.17 (1.16, 1.19) <0.0001 | 1.12 (1.11, 1.14) <0.0001 |
| Diversity of concepts | 1.17 (1.13, 1.22) <0.0001 | 1.08 (1.04, 1.13) 0.0003 |
| Diversity of content | 1.19 (1.16, 1.22) <0.0001 | 1.11 (1.08, 1.14) <0.0001 |
| Interactivity quality | 1.19 (1.17, 1.22) <0.0001 | 1.13 (1.11, 1.16) <0.0001 |

The data is represented by OR (95% CI) P value, OR per 1-point increase in read scale score.

Non-adjusted model adjust for: None

Adjusted Model adjust for: district; kindergarten; household registration of children; child age; child gender; parental age; parental gender; education level; employment status; annual family income; marital status; number of children; average outdoor time; average screen time; CES-D; smoking status; alcohol intake status

Supplementary Table 7 Associations between shared reading and emotional/behavioral problems and prosocial behavior in preschool children

| **Exposure** | **Non-adjusted Model** | **Adjusted Model I** | **Adjusted Model II** |
| --- | --- | --- | --- |
| **Emotional/behavioral problems** |  |  |  |
| **Read scale** | -0.28 (-0.30, -0.26) <0.0001 | -0.24 (-0.26, -0.22) <0.0001 | -0.17 (-0.19, -0.16) <0.0001 |
| Quantity | -0.41 (-0.44, -0.38) <0.0001 | -0.33 (-0.37, -0.30) <0.0001 | -0.25 (-0.28, -0.22) <0.0001 |
| Diversity of concepts | -0.46 (-0.57, -0.35) <0.0001 | -0.47 (-0.58, -0.37) <0.0001 | -0.38 (-0.47, -0.28) <0.0001 |
| Diversity of content | -0.47 (-0.54, -0.40) <0.0001 | -0.37 (-0.44, -0.31) <0.0001 | -0.29 (-0.35, -0.22) <0.0001 |
| Interactivity quality | -0.55 (-0.61, -0.49) <0.0001 | -0.47 (-0.53, -0.42) <0.0001 | -0.31 (-0.36, -0.26) <0.0001 |
| **Prosocial behavior** **adequate** |  |  |  |
| **Read scale** | 0.13 (0.12, 0.14) <0.0001 | 0.12 (0.12, 0.13) <0.0001 | 0.11 (0.10, 0.12) <0.0001 |
| Quantity | 0.18 (0.17, 0.20) <0.0001 | 0.18 (0.17, 0.19) <0.0001 | 0.16 (0.14, 0.17) <0.0001 |
| Diversity of concepts | 0.23 (0.18, 0.27) <0.0001 | 0.18 (0.13, 0.23) <0.0001 | 0.20 (0.16, 0.25) <0.0001 |
| Diversity of content | 0.23 (0.21, 0.26) <0.0001 | 0.20 (0.18, 0.23) <0.0001 | 0.20 (0.17, 0.22) <0.0001 |
| Interactivity quality | 0.25 (0.23, 0.28) <0.0001 | 0.24 (0.22, 0.26) <0.0001 | 0.21 (0.19, 0.24) <0.0001 |

The data is represented by β (95% CI) P value, β is per one-point increase in the read score. .

Non-adjusted model adjust for: None

Adjusted Model I adjust for: district; kindergarten; household registration of children; child age; child gender; parental age; parental gender; education level; employment status; annual family income; marital status; number of children

Adjusted Model II adjust for: All variables of Model I and average outdoor time; average screen time; CES-D; smoking status; alcohol intake status
